# Canagliflozin inhibits growth of hepatocellular carcinoma via blocking glucose-influx-induced β-catenin activation

**DOI:** 10.1038/s41419-019-1646-6

**Published:** 2019-05-29

**Authors:** Man-Hsin Hung, Yao-Li Chen, Li-Ju Chen, Pei-Yi Chu, Feng-Shu Hsieh, Ming-Hsien Tsai, Chih-Ting Shih, Tzu-I Chao, Chao-Yuan Huang, Kuen-Feng Chen

**Affiliations:** 1 Division of Medical Oncology, Department of Oncology, Taipei Veterans General Hospital, Taipei, Taiwan; 2 School of Medicine, National Yang-Ming University, Taipei, Taiwan; 3 Laboratory of Human Carcinogenesis, Center for Cancer Research, National Cancer Institute, Bethesda, MD USA; 4 Department of Surgery, Changhua Christian Hospital, Changhua, Taiwan; 5 School of Medicine, Kaohsiung Medical University, Kaohsiung, Taiwan; 6 Department of Medical Research, National Taiwan University Hospital, Taipei, Taiwan; 7 Department of Pathology, Show Chwan Memorial Hospital, Changhua, Taiwan; 8 Institute of Biopharmaceutical Sciences, National Yang-Ming University, Taipei, Taiwan; 9 Division of Radiation Oncology, Department of Oncology, National Taiwan University Hospital and National Taiwan University College of Medicine, Taipei, Taiwan; 10 Department of Medical Imaging Radiological Technology, Yuanpei University, Hsinchu, Taiwan

**Keywords:** Cancer metabolism, Targeted therapies, Liver cancer

## Abstract

Accelerated glucose metabolism is critical in hepatocarcinogenesis, but the utilities of different glucose transporter inhibitors in treating hepatocellular carcinoma (HCC) remain largely uncharacterized. In this study, we examined a collection of glucose transporter inhibitors and found differential anti-HCC effects among these compounds. Canagliflozin (CANA), phloretin, and WZB117 decreased cellular glucose influx, but only CANA showed potent growth inhibition in HCC, which indicated a glucose-independent anti-HCC mechanism. Notably, we found that CANA treatment significantly downregulated the expression of β-catenin in HCC cells in. By co-treating cells with cycloheximide and MG-132, we proved that CANA promoted proteasomal degradation of β-catenin protein by increasing phosphorylation of β-catenin, and CANA-induced inactivation of protein phosphatase 2A was identified being responsible for this effect. Moreover, using Huh7 xenografted tumor model, CANA treatment was shown to delay tumor growth and improved the survival of HCC bearing mice. Our study highlights the unique dual β-catenin-inhibition mechanisms of CANA, which may provide new thoughts on treating HCC patient with concurrent diabetes, and, furthermore, on developing novel treatment targeting metabolic reprogram and/or WNT/β-catenin signaling in HCC.

## Introduction

Hepatocellular carcinoma (HCC), one of the most fatal human malignant diseases worldwide, is characterized by complex and heterogeneous factors^1^. The vast number of etiologies involved in tumor formation leads to a huge diversity of molecular signatures in HCC patients and poses a major challenge in successful treatment. Sorafenib and regorafenib are currently approved for patients with advanced HCC^2,3^, but the response rate and the actual survival improvement to all of above-mentioned treatments are limited. Therefore, novel treatments to improve outcomes of HCC patients are sorely needed.

Metabolic reprograming, one of the hallmarks of cancer, describes changes in uptake and utilization of different nutrients by cancer cells to attain high growth and proliferation rates^4^. Among all the metabolic alterations, accelerated glucose metabolism may be the best-recognized example. By preferentially expressing isomers of glucose transporters, such as glucose transporter (GLUT) 1, key enzymes, such as hexokinase 2, and pyruvate kinase M2 (PKM2), cancer cells modulate and hijack the whole process of glucose metabolism to balance their inefficient glucose utilization, aerobic glycolysis (also known as Warburg effect), and high anabolic demands^4,5^. Notably, the reprogrammed glucose metabolism cascade not only provides cancer cells with energy, it also promotes the function of many oncoproteins, and drives the survival and progression of cancer cells^6,7^. Meanwhile, oncogenic signaling, such as Akt, also can help progression of reprogrammed glucose metabolism via increasing the expression of glucose transporters on the cell surface or upregulating the function of the enzymes throughout the glucose metabolic cascade^8,9^. This bi-directional interplay highlights the important roles of glucose metabolism in the process of oncogenesis, and makes targeting metabolic alterations an attractive strategy for cancer treatment.

In HCC, aberrant expression of glucose transporters is one of the major features that distinguish tumors and normal tissue. Because one of the major functions of the liver is to maintain the homeostasis of circulating glucose level in the human body, which means that normal differentiated hepatocytes not only consume glucose but also export it (glycogenesis). Bi-directional glucose transporters, such as glucose transporter (GLUT) 2^10,11^, are predominantly expressed in normal liver tissue. Conversely, HCC cells mainly take up glucose to maintain their survival, and overexpress other glucose transporters, such as GLUT1 and GLUT3 that only allow glucose influx^12^. In addition to GLUTs, another energy-dependent sodium/glucose cotransporter 2 (SGLT2) has also been found in liver tissues, but its function in the liver or HCC tissue has not yet been fully disclosed. It has been reported that GLUT1 inhibitors exert some anti-HCC cancer effects^4^, but little is known about other GLUT- or SGLT-inhibitors.

Canagliflozin (CANA) is a novel SGLT2 inhibitor that proved to lower patients’ glycemic level via reducing renal glucose reabsorption^13^. Notably, besides glycemic control, some pre-clinical studies showed that CANA inhibited the growth of prostate, lung and pancreatic cancer^14^, but the effects of CANA or other gliflozins in HCC have not yet been clarified. Furthermore, in Villani’s work^14^, we noted that the doses of CANA required for reducing the cologenic survival of A549 and H1299 were much lower than that for suppressing short-term cell proliferation, which indicates that CANA has a role in inhibiting the cancer-initiating (or stemness) features. Since aberrant activation of WNT/β-catenin signaling is one of the major and commonly observed genetic alterations in the HCC population and its role in contributing to cancer stemness is well characterized, we initiated the present study to uncover the function and mechanism of CANA and other glucose transporter inhibitors in HCC, with particular focus on β-catenin signaling. Surprisingly, we found that CANA exerts two mechanisms to suppress β-catenin activation in an SGLT2-independent manner. Importantly, through inhibiting β-catenin-related oncogenic signaling, we show that CANA treatment potently inhibits the activation of β-catenin signaling and confers inhibition of HCC growth in vitro and in vivo.

## Results

### Canagliflozin treatment inhibits the maintenance of HCC cells and HCC stem cells

**Fig. 1:**
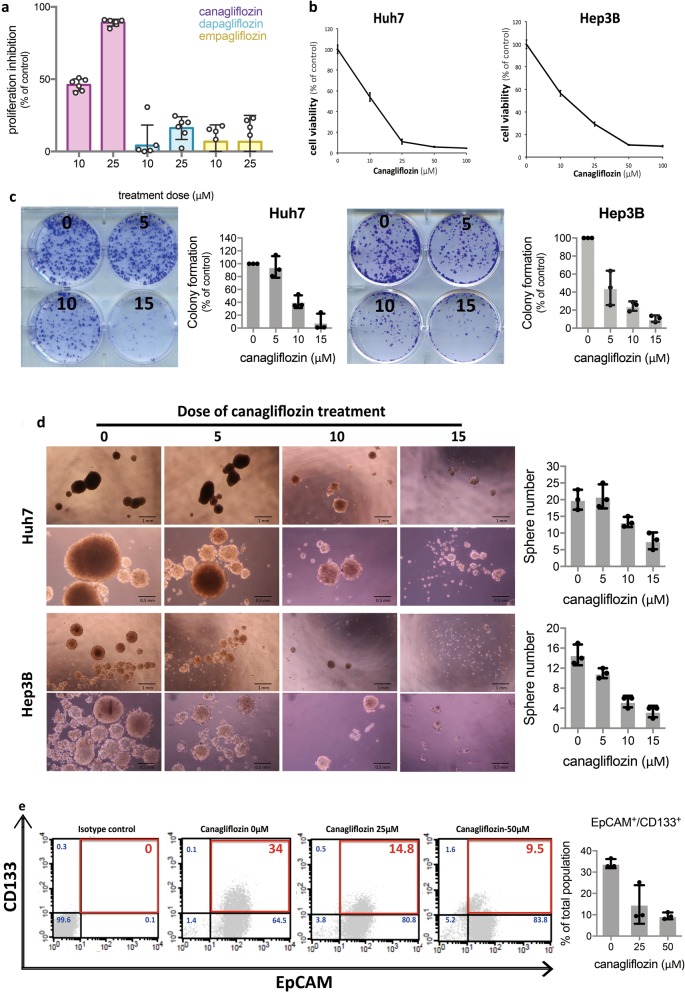
CANA treatment suppressed the growth and stemness properties of HCC cells. **a** SGLT2 inhibitors produced differential anti-HCC effects. The effects of three different SGLT2 inhibitor on affecting the prolieferation of Huh7 cells were examined by MTT assay (*n* = 6). Bar: mean, error bar: S.E. **b** CANA treatment dose-dependently inhibited the viabilities of HCC cells. HCC cells were pre-fasting and treated with CANA at indicated doses for 48 h in low glucose-containing medium and examined by MTT assay (*n* = 6). Point: mean, error bar: S.E. **c** CANA significantly suppressed the generation of HCC colonies. Left: representative images of colony formation; right: quantified results. *N* = 3, Bar: mean, error bar: S.E. **d** CANA treatment reduced the formation of hepatospheres in a dose-dependent manner. *N* = 3. Representative images of sphere formation at different doses and magnifiers (left); quantified results (right) are shown. Bar: mean, error bar: S.E. **e** Percentages of HCC cells with positive expression of CD133 and EpCAM were detected by flow cytometry. *N* = 3. Representative histogram of flow cytometry (left) and quantified results (right) are shown. Bar: mean, error bar: S.E.

To investigate the effects of CANA in HCC, we first exposed Huh7 and Hep3B cells to various doses of CANA and examined the cells by MTT, colony formation assay and hepatoshpere formation assay. As shown in Fig. 1a–c, CANA treatment suppressed the viabilities and colony forming abilities of HCC in a dose-dependent manner. Interestingly, we noted that CANA also suppressed the anchorage growth of HCC cells (Fig. 1d), which implied that the stemness properties of HCC were affected. To further prove this finding, we used two well-recognized markers, EpCAM and CD133, to characterize the subpopulation of HCC stem cells, and found that CANA treatment significantly decreased the proportions of CD133- and EpCAM-positive HuH7 cells (Fig. 1e).

### Canagliflozin targets HCC through an SGLT2-independent mechanism

**Fig. 2:**
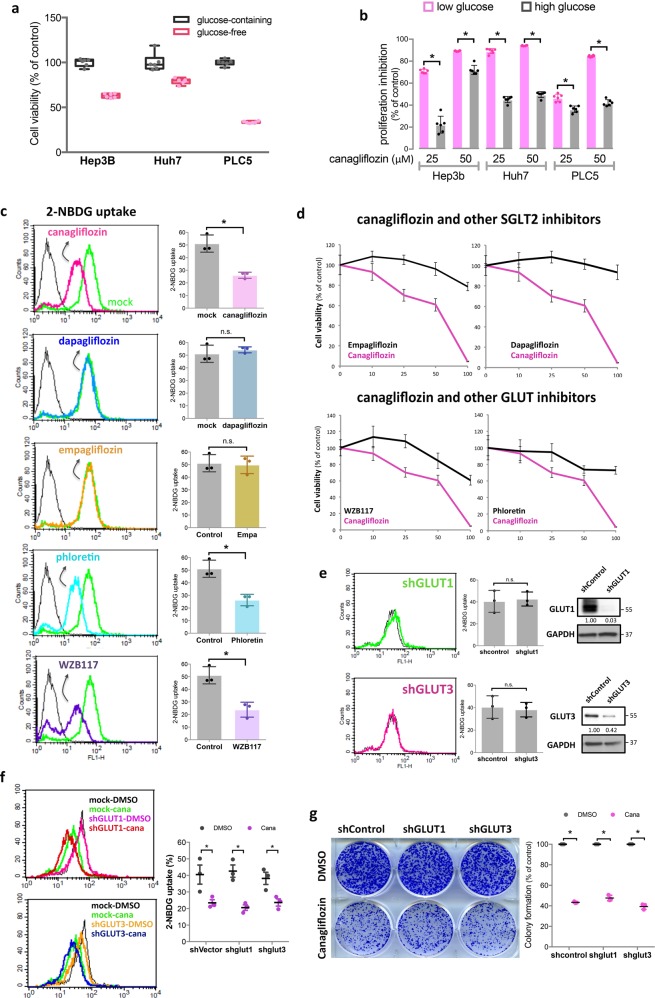
CANA exerts glucose-blockage-independent mechanism against HCC. **a** Glucose-depletion environment compromised the proliferation of HCC cells. Cellular viabilities of the indicated cell lines in glucose-containing and glucose-free environments were measured by MTT assay. Bar, mean; error bar, S.E. **b** The anti-HCC effects of CANA were diminished in a high-glucose-containing environment. HCC cells maintained in low-glucose (12.5 mM) and high-glucose (25 mM)-containing media were treated with CANA for 48 h, and assessed by MTT. Bar, mean; error bar, S.E; *P < 0.05. **c** CANA and GLUT inhibitors, not other SGLT2 inhibitors, reduced glucose uptake of HCC cells. A 2-NBDG glucose uptake assay was conducted to estimate the effects of five different glucose transporter inhibitors, including CANA, dapagliflozin, and empagliflozin (the SGLT2 inhibitor class), and phloretin and WZB117 (the GLUT inhibitor class), on blocking glucose uptake by Huh7 cells. Representative histograms from flow cytometry testing the indicated compounds (left panel) showed that CANA, phloretin and WZB117 reduced cellular glucose uptake (peak of 2-NBDG shifted to the left), but dapagliflozin and empagliflozin did not (peak of 2-NBDG overlapped with that of mock). Results of each compound in triplicated tests were quantified and are shown on the right side. **d** Comparison of the anti-HCC effects of CANA (pink) and the four other glucose inhibitors (black). *N* = 3. Point, mean; error bar, S.E. **e** Knockdown of GLUT1 or GLUT3 was not sufficient to reduce cellular glucose uptake significantly. Left panel shows the representative 2-NBDG uptake histograms of GLUT1- or GLUT3-silencing cells, the middle panel shows the results of triplicated tests, and the right panel represents the western blot of GLUT1 and GLUT3 expression in these mock and genetically manipulated cells. The numbers listed below each band indicated its relative strength. **f**, **g** The effects of CANA on reducing glucose uptake (**f**) and growth inhibition (**g**) were not significantly affected by either GLUT1- or GLUT3- knockdown. Representative histogram of 2-NBDG uptake and colony formation assay are shown on the left side of each subfigure, and the quantified results are shown on right side. *N* = 3, Bar, mean; error bar, S.E

Because the best-known function of CANA is blocking SGLT2-mediated glucose uptake, we began our investigation on the anti-HCC mechanism of CANA by investigating whether glucose influx affected the viability of HCC cells, and, if so, how it related to the anti-HCC effects of CANA. We found that, in line with exposing cells to CANA treatments (Fig. 1a), lowering the glucose content in the culture medium significantly decreased the viabilities of HCC cells (Fig. 2a), while, increasing glucose exposure diminished the effects of CANA. These observations suggested that blocking glucose influx of HCC cells plays a role in mediating the effects of CANA. According to the literature, at a higher dose, CANA may affect not only SGLT2, but also other SGLT and glucose transporters^15,16^, particularly glucose transporter 1 (GLUT1), a frequently overexpressed glucose transporter in HCC. To pinpoint the true target responsible for the effects of CANA, we enrolled two other SGLT2 inhibitors, dapagliflozin and empagliflozin, and two GLUT1 inhibitors, phloretin and WZB117, and compared their effects with CANA. Using the 2-NBDG assay, we observed that CANA treatment significantly reduced the glucose uptake of Huh7 cells (the peak of fluorescence, indicated in pink in the first panel of Fig. 2c, shifted to left). Notably, while phloretin and WZB117 inhibited glucose uptake to a similar extent as CANA, neither of the two SGLT2 inhibitors inhibited glucose uptake (Fig. 2c). Since dapagliflozin and empagliflozin are known to have more specific and potent SGLT2 inhibition than CANA, the results of glucose uptake assay suggested that CANA might work through GLUT-, not SGLT2, inhibition. To test this hypothesis, we used MTT to examine the growth-inhibitory abilities of these compounds in HCC. As expected, neither empagliflozin nor dapagliflozin showed any effects in HCC (upper panel of Fig. 2d). However, though phloretin and WZB117 treatments were shown to inhibit HCC cell growth at higher doses, the extent of the effects were minor in comparison with CANA (lower panel of Fig. 2d), which indicated that GLUT1 inhibition might not fully explain the effects of CANA. To exclude the possibility of insufficient GLUT-inhibition by phloretin and WZB117, we used a shRNA-mediated approach to knockdown the two most important GLUTs, GLUT1 and GLUT3, in HCC. Surprisingly, we found that knockdown of either GLUT1 or GLU3 did not produce any attenuation of glucose uptake in Huh7 cells (Fig. 2e). More importantly, the effects of CANA on blocking glucose influx and inhibiting HCC growth were not affected in either GLUT1- or GLUT3-silenced cells (Fig. 2f, g). The above findings suggest first that CANA may block glucose influx through inhibiting multiple GLUTs, not SGLT2, and, second that inhibition of glucose influx cannot fully explain the anti-HCC effects of CANA.

### Canagliflozin attenuates glucose influx-induced β-catenin signaling activation through promoting its proteasome degradation

**Fig. 3:**
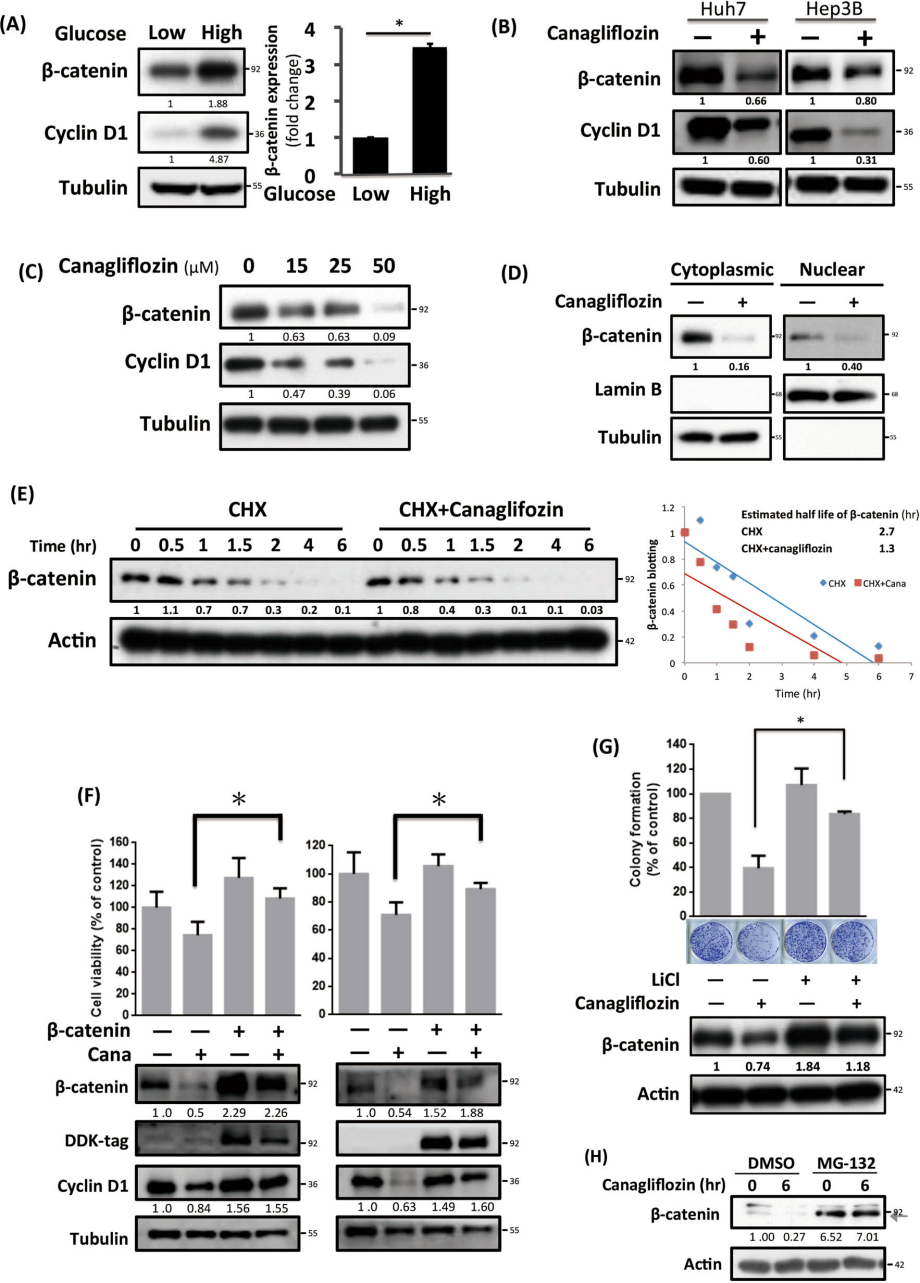
CANA inhibited β-catenin activation to suppress HCC growth. **a** A high-glucose-containing environment induced activation of β-catenin signaling. The expression of β-catenin and cyclin D1 was determined by western blot (left). Fold changes of β-catenin expression (right) were calculated by comparing the β-catenin expression of cells exposed to high (25 mM) versus low glucose conditions (12.5 mM). The intensity of each band was normalized with its loading control from individual experiments. *N* = 3. (**b**) CANA treatment suppressed β-catenin activation in Huh7 and Hep3B cells. HCC cells were treated with or without CANA 25 μM for 24 h and harvested for western blot. Representative western blot images and the intensities (normalizing to loading control) were shown. *N* = 3. **c**, **d** CANA suppresses β-catenin activation in HCC. The effects of CANA on Huh7 cells at the indicated doses (**c**) or at indicated cell fractions (**d**) were examined by western blot. *N* = 3. Relative intensity of each band was listed below. CANA treatment enhanced degradation of β-catenin. Huh7 cells treated with cyclohexamide (CHX) alone or with CANA were harvested at the indicated times and analyzed by western blot. After normalizing with the loading control, the half-life of β-catenin was estimated (right). **f**, **g** β-catenin determines the anti-HCC effects of CANA. The effects of β-catenin on suppressing HCC growth and upregulating cyclin D1 were diminished by ectopic overexpression of β-catenin (**f**), or by co-treatment with the β-catenin activator, LiCl (**g**). Relative intensity of each band was presented. **h** Canagliflozin-induced downregulation of β-catenin expression was reversed by inhibition of proteasome. The expressions of β-catenin (indicated by arrow) under treatment of canagliflozin and/or MG-132, a proteasome inhibitor, were detected by western blot analysis. The number below each band indicated the relative protein intensity under each treatment

As we shown previously that CANA attenuated the maintenance of HCC stem cell, we were interested to known whether the anti-HCC effects of CANA could be better explained from this aspect. We examined the effects of environmental glucose content on WNT/β-catenin signaling. As shown in Fig. 3a, the expression of β-catenin and its downstream protein, cyclin D1, rose significantly in high-glucose condition (Fig. 3a), and CANA treatment counteracted with such changes in Huh7 and Hep3B cells (Fig. 3b). Furthermore, such changes progressed with increasing CANA exposure (Fig. 3c). Because β-catenin functions as a transcription factor, we further showed that CANA treatment decreased the nuclear translocation of β-catenin (Fig. 3d). Next, by pretreating cycloheximide and tracing the sequential change of β-catenin, we observed that the half-life of β-catenin was significantly shortened in CANA-treated cells, implying CANA affected the protein stability of β-catenin (Fig. 3e). Next, we used two different strategies to confirm the significance and necessity of β-catenin associated with the anti-HCC effects of CANA. First, we generated β-catenin ectopically expressed cells and showed that the effects of CANA on inhibiting β-catenin/cyclin D1 and suppressing HCC growth were abolished by overexpression of β-catenin in both Huh7 and Hep3B cells (Fig. 3f). Furthermore, co-treatment of cells with LiCl, a known β-catenin activator^17^, diminished the effects of CANA (Fig. 3g).

### Canagliflozin promotes the degradation of β-catenin via inhibiting PP2A-mediated dephosphorylation of β-catenin

**Fig. 4:**
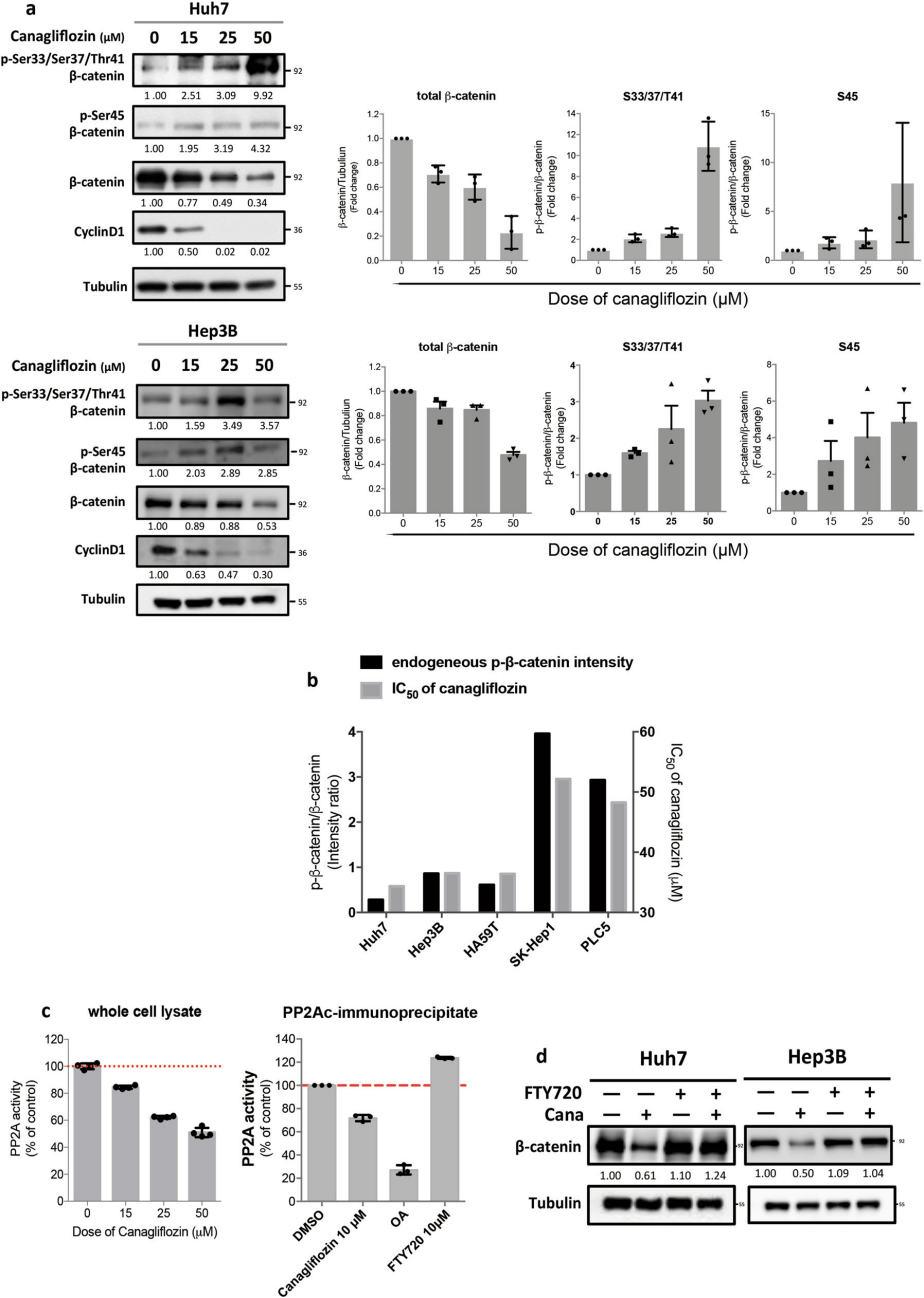
CANA promoted β-catenin degradation through inhibiting PP2A-mediated dephosphorylation of β-catenin. **a** CANA treatment upregulated the expression of p-β-catenin and inhibited β-catenin/cyclin D1 signaling in a dose-dependent manner. The relative signal intensity was listed below each band, and the average changes of p-β-catenin in triplicated tests were shown on right. **b** Sensitivity to CANA treatment was associated with endogenous β-catenin activity in cancer cells. The endogenous β-catenin activity was estimated by the baseline expressions of p-β-catenin to total β-catenin (left *y*-axis) in four different HCC cell lines, namely Huh7, Hep3B, HA59T and PLC5, and SK-Hep1 cell, an epithelial adenocarcinoma cell line derived from the ascites of a HCC patient. The IC_50_ was determined by the results of MTT (right *y*-axis). *N* = 6. See also Supplement Fig. 1a, b. **c** CANA treatment inhibited the activities of PP2A in HCC. After exposing Huh7 cells to the indicated treatments for 24 h, the PP2A activities of whole cell lysate were determined. Meanwhile, the effects of CANA were also tested directly on PP2Ac-immunoprecipitant isolated from Huh7 cells. Okadaic acid (OA), PP2A inhibitor, was used as a positive control, and FTY720, PP2A activator, as a negative control. *N* = 3. Bar, mean; error bar, S.E. **d** Co-treatment with the PP2A activator diminished the inhibitive effects of CANA on β-catenin. Cells treated with CANA and/or FTY720 were analyzed by western blot. *N* = 3, representative images with quantified intensity are shown

It is known that β-catenin mainly undergoes proteasomal degradation^18^; we used MG-132, a proteasome inhibitor, and showed that the CANA-mediated downregulation of β-catenin was attenuated by MG-132 (Fig. 3h). Because the amino-terminal serine/threonine phosphorylation of β-catenin is the key to the initiation of its proteasomal degradation machinery^18^, we continued to examine the expression of p-β-catenin at various serine/threonine sites. As shown in Fig. 4a, CANA treatment dose-dependently increased the expression of p-β-catenin at Ser45 and Ser33/Ser37/Thur41, which corresponded to the changes of β-catenin and cyclin D1 expression. Furthermore, we found that the sensitivity of CANA is linked with the endogenous β-catenin activity among different cancer cell lines. Notably, the IC50 of CANA were relatively lower in cells with lower p-β-catenin to total β-catenin expressions than that of cells with higher p-β-catenin expression (Fig. 4b, Supplement Fig. 1a, b). Next, we investigated how CANA affected β-catenin at so many different phosphorylation sites. Previously, protein phosphatase 2A (PP2A) was characterized to regulate β-catenin phosphorylation^19^. To clarify the potential role of PP2A in mediating the effects of CANA, we first examined whether PP2A-silencing cells exhibited a similar phenomenon. As expected, we observed that PP2A-knockdown led to upregulation of p-β-catenin at Ser45 and Ser33/Ser37/Thur41 and downregulation of the expressions of total β-catenin and cyclin D1 (Supplement Fig. 1c). Furthermore, we showed that CANA treatment decreased the activity of PP2A in whole cell lysates and PP2Ac-immunoprecipited lysates, indicating that CANA may directly interact with the PP2A-β-catenin complex (Fig. 4c). To validate the role of PP2A, we exposed HCC cells to a PP2A enhancer, FTY720, and found that CANA-induced downregulation of β-catenin was reversed (Fig. 4d).

### Canagliflozin suppresses in vivo tumor growth and prolongs the survival of tumor-bearing mice via inhibiting PP2A/p-β-catenin

**Fig. 5:**
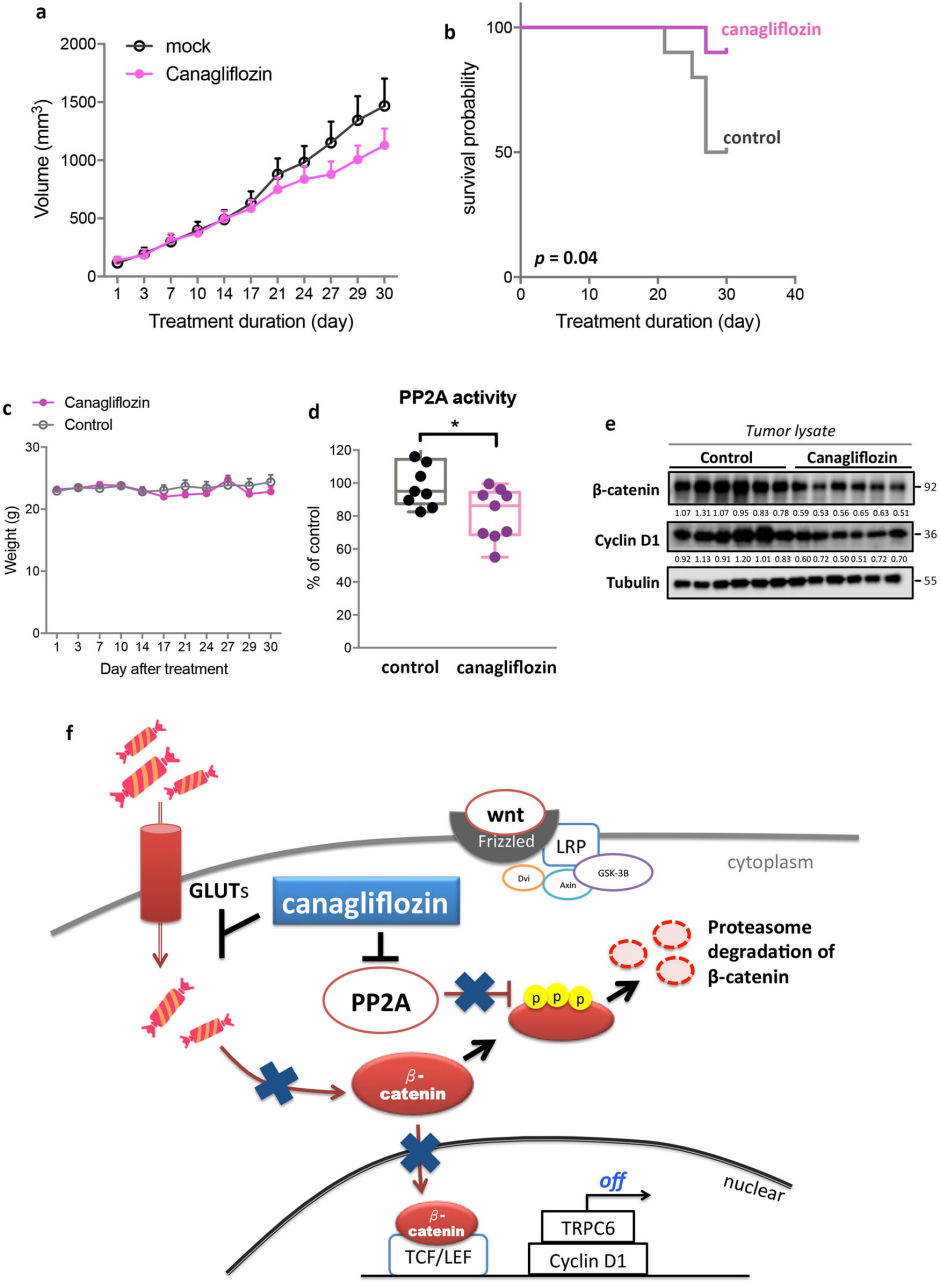
CANA treatment suppressed the growth of tumors and prolonged the survival of tumor-bearing mice via inhibiting PP2A/β-catenin activation in HCC. **a** CANA treatment reduced the growth rates of Huh7-xenograft tumors. Point: mean; error bar, S.E. **b** CANA treatment significantly prolonged the survival of tumor-bearing mice. The survival event was determined by the recorded death of mice or an estimated tumor size of larger than 1500 mm^3^. The *p* value was determined by log-rank test. **c** The average of body weight of mice during treatment. **d**, **e** CANA treatment inhibited the PP2A activity and expression of β-catenin in vivo. Tumors from each treatment arm were harvested and analyzed by PP2A activity (**d**) and western blot (**e**). *p < 0.05. The number below each band indicated its relative intensity. **f** The schema illustrates the dual β-catenin-inhibition mechanism of CANA. Through inhibiting PP2A activity, CANA upregulated the expression of p-β-catenin and subsequently promoted the proteasomal degradation of β-catenin. Conversely, CANA treatment reduced GLUT-mediated glucose-influx in HCC, which further blocked β-catenin activation

To validate our findings in vivo, we used a Huh7 xenografted tumor model. Because little information is available about the appropriate dose and safety of CANA treatment in tumor-bearing mice, we applied a two-stage dosing schedule. In the first 10 days, we fed mice with CANA at 100 mg/kg/day, and after confirming good tolerance in general, we increased the drug dose to 300 mg/kg/day and followed the survival and tumor growth until death or day 30. CANA treatment suppressed the growth of tumors, particularly during the second dose period, and significantly prolonged the survival of mice (Fig. 5a, b). As shown in Fig. 5c, mice tolerated the whole course of treatment quite well; there was no weight change during the first dose period, and only mild reversible weight loss (<10%) was observed during the second dose period. Furthermore, we did not observe significant changes of the glycemic level, the function of kidney and liver, and whole blood cell counts in mice treated with CANA as comparing to mock-treated mice (Supplement Fig. 2). To confirm the biological effects of CANA treatment, we examined tumor lysates after completing all the experiments and found that the tumor lysates presented with decreasing PP2A activity and β-catenin expression (Fig. 5d, e). To sum up, we found that CANA exerts dual function when inhibiting β-catenin signaling in HCC; first, CANA blocks glucose-influx-induced β-catenin upregulation, and, second, CANA directly inhibits PP2A to promote the proteasome-dependent degradation of β-catenin (Fig. 5f).

### β-catenin upregulation is a tumor-specific event in HCC patients

**Fig. 6:**
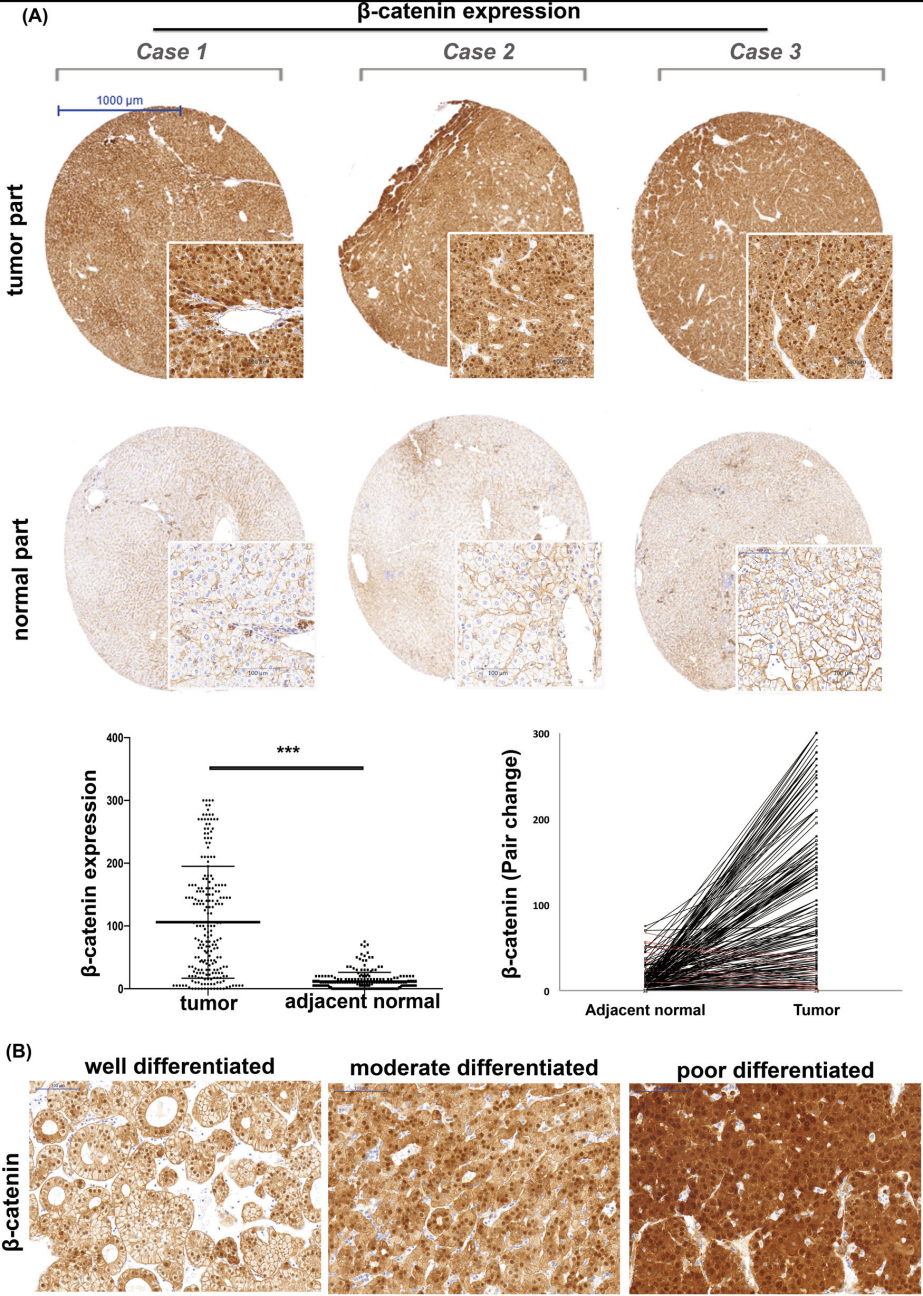
Overexpression of β-catenin in HCC clinical samples is frequent and highly tumor-specific, and is associated with poor tumor differentiation. **a** Representative IHC images of the β-catenin expression in the tumor and paired non-tumor part are shown in the upper panel. The lower left panel shows the average distribution of β-catenin expression in tumors and adjacent liver tissue. The lower right panel represents the paired comparison of tumor and non-tumor parts (black line: patients with higher β-catenin in tumor; red line: patients with the reverse trend). *N* = 216. **b** The expression of β-catenin increased alone with deteriorated tumor differentiation. Representative IHC images of β-catenin expression in tumorswith well, moderate and poor tumor differentiation

To understand whether β-catenin is a feasible target for the development of anti-HCC therapy, we examined clinical tumor samples obtained from 216 HCC patients. As shown in Fig. 6, we found that the cytoplasmic and/or nuclear staining of β-catenin was only significant in the liver tumors, not in the adjacent non-tumor parts (*p* = 0.015), and the upregulation of β-catenin was associated with poor tumor differentiation (Fig. 6b, *p* = 0.015).

## Discussion

Reprograming of glucose metabolism is a well-recognized hallmark of HCC and other cancers, but understanding how extensively this metabolic adaption impacts cancer cells is still an ongoing task. In this work, we highlighted the effects of accelerated glucose influx on upregulating β-catenin signaling in HCC and characterized the effects of a glucose inhibitor, CANA, in blocking this glucose-influx mediated β-catenin activation (Figs. 1–3). The role of WNT and β-catenin in regulating metabolic homeostasis was first suggested because of the observations that genetic mutations of WNT and β-catenin signaling affected the susceptibility to obesity and diabetes, and loss of β-catenin protected rodents from high-fat-diet-induced fatty liver, obesity and insulin resistance^20,21^. Later on, the role of the WNT signaling network in regulating metabolic reprograming, particularly involving glucose utilization, in cancer cells drew increasing attention. Activation of WNT/β-catenin signaling was shown to promote aerobic glycolysis in cancer cells via suppressing mitochondrial respiration, inducing the activity of pyruvate carboxylase and increasing the expression pyruvate dehydrogenase kinase 1^22,23^. On the other hand, c-Myc, a well-recognized downstream factor of β-catenin, transcriptionally controls many essential elements driving cancer metabolic reprograming, such as forkhead transcription factors, GLUT1, and PKM2^24,25^. Interestingly, in the present work, we showed that the crosstalk of β-catenin and glucose metabolism could work in the opposite direction; exposure to a high-glucose-containing culture medium significantly enhanced the expression of β-catenin and cyclin D1 downstream of β-catenin, in HCC cells (Fig. 3a). Chouhan et al. showed that a high-glucose environment suppressed the function of DKK4 (a Wnt antagonist) and increased β-catenin acetylation^26,27^. The observations from the current work and Chouhan’s work suggest that WNT and β-catenin not only promote reprograming of glucose metabolism, but increasing glucose uptake of cancer cells also leads to activation of β-catenin signaling. Taken together, these results suggest that WNT/β-catenin signaling has a central role in regulating metabolic reprogramming of cancer cells.

According to several large-scale genetic studies, aberrant activation of WNT/β-catenin-related signaling is one of the most common genetic predispositions in the HCC population, which makes WNT/β-catenin signaling an attractive target for designing novel anti-HCC treatments^28,29^. The canonical WNT signaling cascade is initiated when Wnt ligands bind to the Frizzled receptors and subsequently uncouple β-catenin from E-cadherin. Once β-catenin is free from E-cadherin, it translocates from the cytosol into the nucleus and forms a complex with its transcriptional coactivators to regulate genes involved in the development, maintenance and stemness of cancer cells^30,31^. If WNT ligands are not present or not able to bind to Frizzled receptors, β-catenin is phosphorylated, ubiquitinated and undergoes proteasome-mediated protein degradation. Different strategies to target the major components of this signaling cascade have been implicated to design novel WNT/β-catenin inhibitors^31^, and some of these novel compounds, such as the anti-Frizzled OMP-18R5^15^, LGK974 that inhibits WNT ligand secretion^32^, and PRI-724 that perturbs the formation of β-catenin-associated transcriptional regulatory complex^33^, are under active investigation in early phase clinical trials. In the present study, we demonstrated that CANA had dual β-catenin-inhibitory mechanisms and demonstrated its anti-HCC effects in vitro and in vivo (Figs. 3–5). Importantly, this anti-β-catenin property of CANA is unique among its class. Similar differential effects against cancer have been observed in breast, colon, and lung cancer cells by Villani et al.^14^ Interestingly, in an earlier phase 3 trial testing dapagliflozin in patients with type 2 diabetes mellitus, an increasing breast and bladder cancer incidence was reported^34^, but such a phenomenon was not observed in trials testing CANA. Because CANA and the other two SGLT2 inhibitors all have the same C-glucoside structure with different modifications at the heteroaromatic ring structure, further structure-activity relationship analysis is needed to clarify the element responsible for the differences.

It is a well-recognized phenomenon, particularly supported by the wide and successful application of 2-deoxy-2[^18^F]fluoro-d-glucose positron-emission tomography scan for early cancer detection, that cancer cells have increased glucose uptake from the early stage of tumor development, which may provide a rationale for using glucose transporter inhibitors against cancer^35^. Several different GLUT inhibitors, like phloretin and its derivative, WZB117, were shown to suppress cell proliferation and induce apoptosis by reducing glucose uptake in liver, renal and lung cancer cells^36,37^. In the present study, we compared the effects of phloretin and WZB117 with CANA. Intriguingly, we found that these two compounds, similar to CANA, could reduce glucose uptake of HCC cells, but neither of them destabilized β-catenin and suppressed HCC growth in the same way as CANA (Fig. 2c, d). The differential effects indicate that CANA utilizes a GLUT-independent mechanism to inhibit in PP2A/β-catenin in HCC. Moreover, when we generated shRNA-mediated GLUT1- and GLUT3-knockdown cells to clarify the necessity of these two proteins for the effects of CANA, surprisingly we found that GLUT1- or GLUT3-silencing alone was not sufficient to induce reduction of glucose uptake in HCC cells (Fig. 2e). This finding has three possible explanations. First, given the critical nature of glucose for cell survival, the expression of different GLUT isomers may be redundant^38^; therefore, single knockdown may be compensated by upregulation of other isomers. Second, CANA may work on more than a single GLUT isomer simultaneously resulting in broader and more comprehensive inhibition of glucose uptake. Third, CANA inactivate β-catenin at the same time; as the literature indicates that β-catenin activation promoted the expression of GLUT isomers, additional β-catenin inhibition may further abolish glucose uptake in cancer cells.

To sum up, the present work highlighted an interesting crosstalk between β-catenin signaling and glucose influx in HCC, and clarified the unique and delicate anti-HCC mechanisms of CANA. Our data provides a new direction for designing and developing novel anti-HCC treatments. More investigations are warranted to clarify how the positive feedback loop linking accelerated glucose metabolism and β-catenin activation impacts the biology of HCC and other cancer cells.

## Materials and methods

### Reagents and antibodies

Canagliflozin, empagliflozin, and dapagliflozin were purchased from Selleck Chemicals (Houston, TX). MG-132, lithium chloride (LiCl), FTY720, WZB117, and phloretin were obtained from Sigma-Aldrich (St. Louis, MO) and Okadaic acid (OA) was from Cayman Chemical (Ann Arbor, MI). For immunoblotting, antibodies against phospho-β-catenin (Ser33/Ser37/Thr41), phospho-β-catenin (Ser45), β-catenin, Cyclin D1, Tubulin, Lamin B, and DDK-tag were purchased from Cell Signaling (Danvers, MA), and Glut1 and GAPDH antibodies were from Abcam (Cambridge, MA). PP2Ac, Glut3, and Actin antibodies were obtained from Millipore (Billerica, MA), Santa Cruz Biotechnology (San Diego, CA), and ProteinTech (Rosemont, IL), respectively. For experiments related to flow cytometry, Monoclonal CD133/2 (293C3)-APC and Mouse-IgG2b-APC antibodies were purchased from Miltenyi Biotech (Bergish Gladbach, Germany), and FITC anti-human CD326 (EpCAM) and Mouse-IgG2b-FITC antibodies were from BioLegend (San Diego, CA).

### Cell culture

The Huh-7 cell line was obtained from the Health Science Research Resources Bank (HSRRB; Osaka, Japan; JCRB0403), HA59T was from the Bioresources Collection and Research Center, Food Industry Research and Development Institute (Hsinchu, Taiwan), and the Hep3B, PLC/PRF/5 (PLC5) and SK-Hep1 cell lines were from American Type Culture Collection (ATCC; Manassas, VA). All cells were maintained in Dulbecco’s modified Eagle’s medium (DMEM) containing 10% fetal bovine serum (FBS), and kept in a 37 °C humidified incubator in an atmosphere of 5% CO_2_ in air. For in vitro experiments, compounds were dissolved in dimethyl sufoxide (DMSO) at the indicated concentrations and added to culture medium to achieve a final concentration of 0.1%. For experiments testing the impact of glucose and those with fasting procedure, we transferred the cells to culture medium containing the indicated concentration of glucose and 5% FBS 4 h before experiments. All of the mediums we used in the present work were purchased from Gibco (Gaithersburg, MD).

### Cell viability and colony formation assay

We used the MTT (3-(4,5-Dimethylthiazol-2-yl)-2,5-diphenyltetrazolium bromide) assay to determine the viabilities of HCC cells after treatments. In brief, 5 × 10^3^ cells were seeded in each well of a 96-well plate and exposed to test compounds at the indicated concentrations in 5% FBS culture medium. After 48 h, 1 mg/ml MTT was added, incubated for 3 h, and analyzed by ELISA reader at 570 nm.

### Colony formation and sphere formation assay

For colony formation assay, Huh7 and Hep3B cells were seeded in a 6-well plate at a density of 1000 cells per well. Fourteen days later, numbers of tumor colonies were determined by 0.5% crystal violet after fixation with 4% of paraformaldehyde solution. For the generation of hepatospheres, Huh7 and Hep3B cells were seed in 24-well ultra-low attachment plates (Corning) at a density of 500 cells per well and grown in serum-free medium. After 7–10 days, these plates were analyzed for hepatosphere formation.

### Glucose uptake assay

We used a 2-deoxy-2-[(7-nitro-2,1,3-benzoxadiazol-4-yl) amino]-D-glucose (2-NBDG) Glucose Uptake Assay obtained from Biovision (Milpitas, CA) to determine the effects of CANA and other glucose transporter inhibitors on blocking cellular glucose uptake. All the procedures were conducted according to the manufacturer’s manual. In brief, Huh7 cells were seeded in DMEM with 10% FBS in 24-well plate at the density of 4 × 10^4^ cells/well. The next day, the culture medium was removed and cells were exposed to the indicated treatments, including 25 μM of CANA, dapagliflozin, empagliflozin, phloretin, and WZB117, within 400 μl 0.5% FBS-containing DMEM medium for 6 h. Next, 2-NBDG-containing glucose uptake mixture was added into each well for another 30 min. The degree of 2-NBDG uptake of each treatment was determined by flow cytometry. Each individual experiment included a mock-treated negative control and a positive phloretin-treated control.

### Gene knockdown

For gene knockdown, we used pLKO.1-puro vector expressing non-targeting control shRNA (pLKO TRC025) or shRNAs targeting GLUT1 (TRCN0000043583) and GLUT3 (TRCN0000436325) to generate Glut1- and Glut3-stable knockdown cells. The shRNA reagents and the recombinant lentiviruses were obtained from the National Core Facility for Manipulation of Gene Function by RNAi (Academia Sinica, Taiwan). The procedure was conducted as described previously^24^. In brief, prior to infection, Huh7 cells were seeded in a 10-cm dish and the lentiviral supernatant was added to the disc at a multiplicity of infection (MOI) of 3. After infection for 24 h, the virus media were replaced with culture media and incubated for another 24 h. Last, the cells were incubated in fresh medium containing 4 μg/ml puromycin for at least 72 h for selection.

### Transfection

β-catenin cDNA (CTNNB1) was purchased from OriGene (Rockville, MD). The cells were transiently transfected with β-catenin for 24 h and exposed to Canagliflozin 25 μM for another 24 h. Lipofetamine 2000 reagent (Invitrogen, Carlsbad, CA, USA) was used for plasmid transfection according to the manufacturer’s instructions.

### PP2A phosphatase activity

We used a PP2A activity kit (Milipore, Billerica, MA) to determine cellular PP2A activity as described previously^39^. In brief, the effects of CANA on affecting PP2A were tested in Huh7 and PP2Ac-immunoprecipitant protein complex harvested from Huh7 cells. After cells exposed to indicate treatments, cell lysates were prepared within a low-detergent lysis buffer, and a specific reaction buffer containing 750 mM phosphopeptide substrate was added. The whole reaction took place for 10 min at 30 °C. The amounts of free phosphate after CANA treatment were determined by the optical density revealed by malachite dye at 650 nm.

### Xenograft tumor growth experiments

We used male NCr athymic nude mice (5–7 weeks of age) obtained from the National Laboratory Animal Center (Taipei, Taiwan) for our in vivo experiments. All study design and experimental procedures using these mice were performed in accordance with protocols approved by the Institutional Laboratory Animal Care and Use Committee of National Taiwan University. In brief, Huh7-xenograft tumors were inoculated subcutaneously on the flank of each mouse with a mixture of 5 × 10^6^ cancer cells suspended in 0.1 ml of serum-free medium containing 50% Matrigel (BD Biosciences, Bedford, MA). For CANA treatment, because of the lack of literature demonstrating the appropriate dose of CANA, we designed a two-stage dosing plan. At the first dose stage, mice were fed with CANA 100 mg/kg/day, this was increased up to 300 mg/kg/day in the second stage. The treatment was started when the xenografted tumors reached 100–150 mm^3^, and stopped when mice died or their tumor reached 1500 mm^3^. Throughout the treatment course, the size of the tumor, the activity and body weight of mice were recorded every other day. At the end of the experiments, the tumors were harvested for western blot and PP2A activity assay. For toxicity experiment, another 10 female NCr athymic nude mice were obtained and were treated with CANA, or vehicle control at 300 mg/kg/day daily by oral gavage for 2 weeks. After planned treatments, mice were transported to Taiwan Mouse Clinic for blood chemistry from complete blood count and biochemistry analysis.

### Clinical samples and immunohistochemistry studies

The study protocol and all the human experiments were approved and conducted according to the guidance of the ethics committee of the Institutional Review Board of Changhua Christian Hospital. For each patient enrolled in the present study, informed consent was obtained at the time of donation in accordance with the Declaration of Helsinki. Primary tumor tissues were obtained from 216 HCC patients receiving surgical resection in Changhua Christian Hospital. For the preparation of tissue array, we took the representative HCC tumors and the adjacent normal tissues, when possible, from each patient and made them into a tissue array. For IHC experiments, the slides containing paraffin-embedded HCC tissue sections were first deparaffinized and rinsed with 10 mmol/L Tris-HCl (pH 7.4) and 150 mmol/L sodium chloride. Then we used methanol and 3% hydrogen peroxide to quench the effects of peroxidase. Before staining, slides were heated at 100 °C in citrate buffer (10 mmol/L, pH 6.0) for 20 min for antigen retrieval. Next, slides were exposed to antibody against β-catenin (BD Transduction Laboratories (Franklin Lakes, NJ), for an hour at room temperature. After carefully wash, we used EnVision Detection Systems Peroxidase/DAB, Rabbit/Mouse Kit (Dako) to detect the signal. For each individual experiment, a prior-confirmed positive sample was included as a positive control and the primary antibody was replaced by PBS as a negative control. All the slides were reviewed by a board-certified pathologist for the intensity and the percentage of stained cells.

### Statistical analysis

All the experiments were conducted in at least triplicate and the results were shown as mean ± standard error (SE). We used student *t*-test, one-way ANOVA and Chi-square to compare the results as appropriate. A *P* value of <0.05 was considered significant. All the statistical analyses were conducted using SPSS software (IBM, version 17.0).

## Supplementary information

Supplement Figure 1-2
