# Supplementary material for: Canagliflozin inhibits growth of hepatocellular carcinoma via blocking glucose-influx-induced β-catenin activation
Source: Cell Death Dis. 2019 May 29;10(6):420. doi: 10.1038/s41419-019-1646-6 (PMC6541593; doi:10.1038/s41419-019-1646-6)

Supplement Figure 1. Sensitivity to canagliflozin links to endogenous activity of β-catenin signaling in HCC

(A) Endogenous expressions of p-β-catenin and β-catenin among different HCC cell lines were examined by western blot.

(B) Dose-related response to CANA treatment was examined by MTT assay. N=6; Bar: mean, Error bar: S.E.

(C) Knockdown of PP2Ac led to upregulation of p-β-catenin and inactivation of β-catenin/cyclin D1 signaling. Numbers represent the intensity of the band (above) after normalizing to the loading control.

Supplement Figure 2. Laboratory examinations of mice treated with CANA. Mice fed with CANA (300 mg/kg/day, n=5) or vehicle control (n=5) for two weeks and, subsequently, underwent detail biochemistry and blood count analysis. Bar: mean; error bar: S.D.

Supplement Figure 1

a

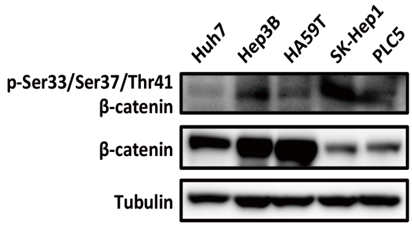

b

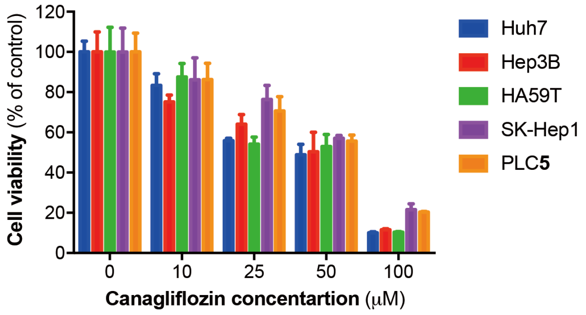

c

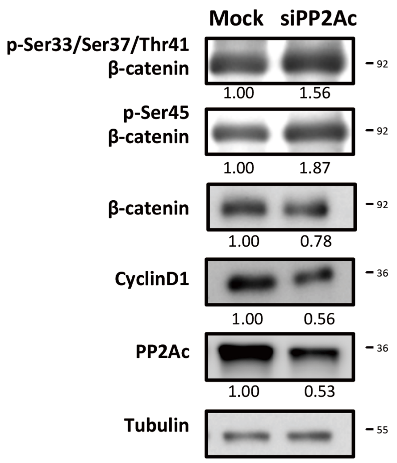

Supplement Figure 2

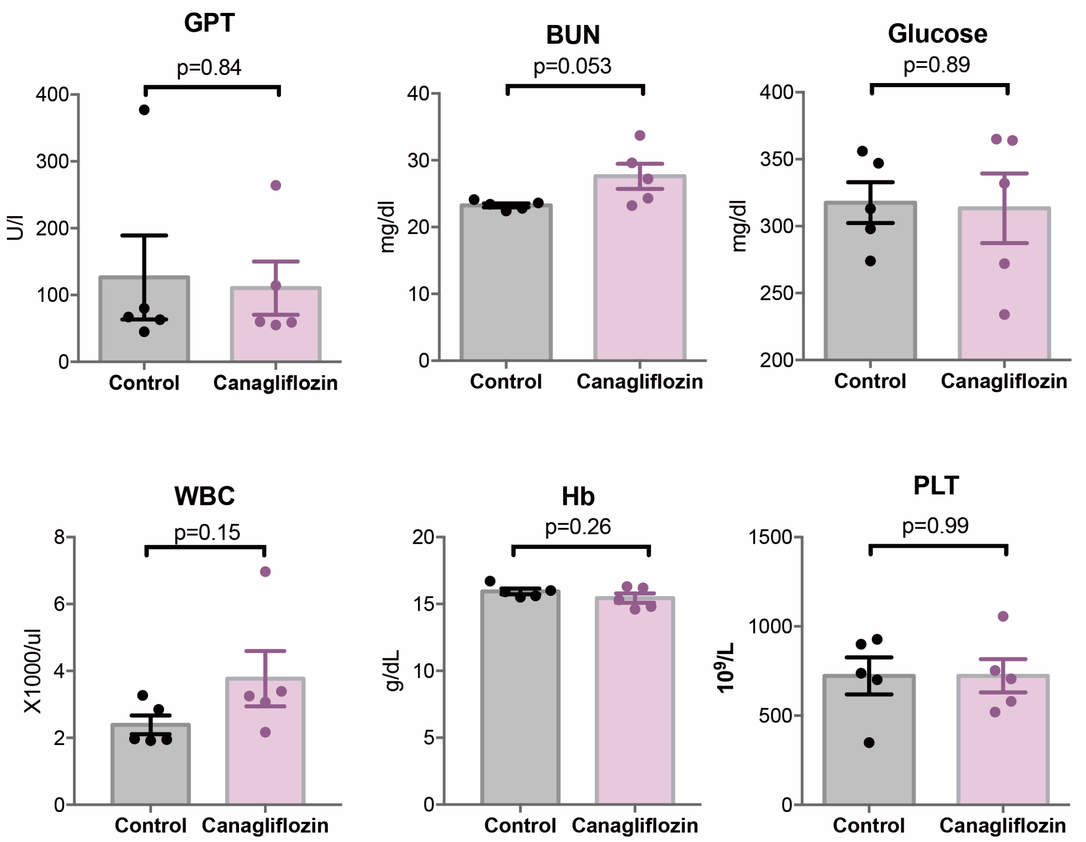

Supplement: Supplementary file 1 — Supplement Figure 1-2 [file 41419_2019_1646_MOESM1_ESM.pdf]
